# First description of the karyotype of a eucharitid wasp (Hymenoptera, Chalcidoidea, Eucharitidae)

**DOI:** 10.3897/CompCytogen.v9i4.5201

**Published:** 2015-09-29

**Authors:** Igor Silva Santos, Jacques Hubert Charles Delabie, Marco Antonio Costa, Cléa Santos Ferreira Mariano, Janisete Gomes Silva

**Affiliations:** 1 Universidade Estadual de Santa Cruz, Departamento de Ciências Biológicas, Ilhéus, Bahia, 45.662-900, Brazil; 2 Instituto Federal de Educação, Ciência e Tecnologia Baiano, Santa Inês, Bahia, 45.320-000, Brazil; 3 Laboratório de Mirmecologia, CEPEC/CEPLAC, Itabuna, Bahia, 45.600-000, Brazil; 4 Universidade Estadual de Santa Cruz, Departamento de Ciências Agrárias e Ambientais, Ilhéus, Bahia, 45.662-900, Brazil

**Keywords:** *Kapala*, parasitoid wasp, Eucharitidae, cytogenetics

## Abstract

The haploid karyotype of *Kapala* sp. (Eucharitidae), a parasite of the Neotropical ant *Dinoponera lucida* Emery, 1901 (Hymenoptera, Formicidae), is reported for the first time. It consists of four metacentric chromosomes. Chromosomes in the family Eucharitidae were unknown so far; therefore, our results confirm that multiple parallel chromosomal fusions have taken place in several lineages within the superfamily Chalcidoidea.

## Introduction

Eucharitidae are a comparatively small family of hymenopteran parasitoid wasps with 423 described species (Noyes 2014). They belong to the superfamily Chalcidoidea, an economically important and extremely diverse group with more than 22,000 described species out of an estimated number of about 500,000 species living today (Gokhman 2013, Noyes 2014). According to Murray et al. (2013), the exclusive association between parasitic wasps within this family and ants started approximately 72 Mya ago. Eucharitids are one of the few wasp groups that were able to break the ants’ communication codes used in kin recognition among colony members via behavioral, morphological, and chemical adaptations (Murray et al. 2013).

Despite the large number of described species, less than 1% of chalcid wasps have been karyotyped so far (Gokhman 2009). Two groups of families used to be recognized within Chalcidoidea based on karyotypes: one group with species showing high chromosome numbers (n=9–11) and a second group with species showing low chromosome numbers (n=3–7) (Gokhman 2005, Gokhman and Gumovsky 2009). Recent studies, however, have revealed an even more complex pattern (Gokhman 2013). For instance, species with lower chromosome numbers (n = 5–7) were found in the families Eurytomidae and Encyrtidae, which initially were placed into the “high- numbered” group (Gokhman 2013). Karyotype evolution in this group has been a matter of discussion for the past decade since chromosome rearrangements are likely to have played an important role in the diversification of parasitoid wasps. However, it is difficult to analyze karyotype changes within a phylogenetic framework since despite recent progress (Munro et al. 2011, Heraty et al. 2013) phylogenetic relationships within Chalcidoidea are still largely unknown. It is noteworthy that depending on the outgroup used, one can arrive at different conclusions based on the same cytogenetic data, as in the case of Eupelmidae (Fusu 2008, Gokhman and Gumovsky 2009).

Wasps of the genus *Kapala* Cameron, 1884 (Hymenoptera: Eucharitidae) are specialized ant parasitoids that are associated with several poneromorph ant genera such as *Ectatomma* F. Smith, 1858, *Gnamptogenys* Roger, 1863, *Typhlomyrmex* Mayr, 1862 (Ectatomminae), *Hypoponera* Santschi, 1938, *Neoponera* Emery, 1901, *Odontomachus* Latreille, 1804, *Pachycondyla* F. Smith, 1858, *Pseudoponera* Emery, 1900 and *Dinoponera* Roger, 1861 (Ponerinae) (Pérez-Lachaud et al. 2006, Buys et al. 2010, Lachaud et al. 2012, Murray et al. 2013, Lachaud and Pérez-Lachaud 2015). So far, only 17 out of more than 60 estimated species have been described within this genus, which is widespread and most commonly collected in the Neotropical region (Heraty 2002, Pérez-Lachaud et al. 2006, Lachaud et al. 2012). Brazil harbors a high diversity of *Kapala* wasps, however, only eight species have been reported to date from this country (Noyes 2014).

In this paper, we present the first description of the karyotype in a eucharitid wasp (*Kapala* sp.) and discuss the importance of these results for the understanding of karyotype evolution in parasitic wasps.

## Material and methods

**Figure 1.**
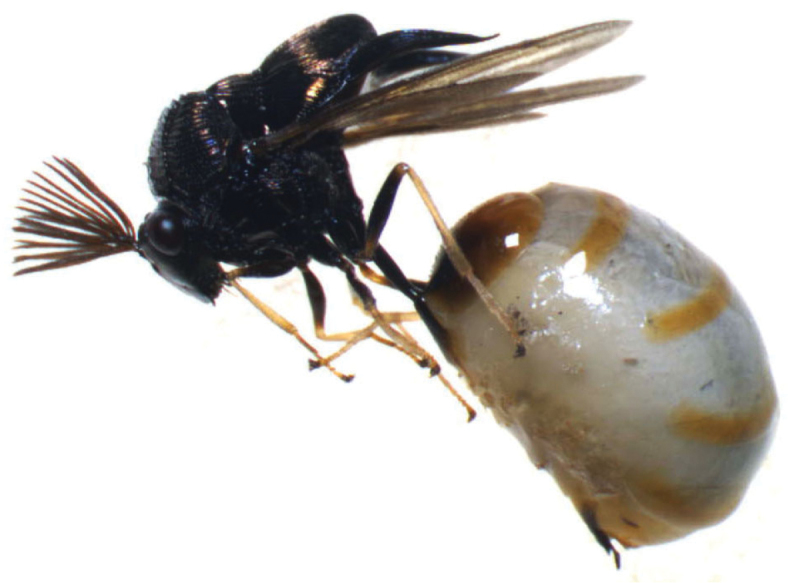
Adult male specimen of *Kapala* sp.

Two specimens of *Kapala* sp. (presumably a new species according to J. Heraty, 2014, in litt.) were found inside the cocoons of *Dinoponera lucida* Emery, 1901 (Formicidae, Ponerinae), a young adult male (Fig. 1) and a female pupa. The adult specimen was deposited at the University of California Riverside Entomology Research Museum, USA. The host colony of *Dinoponera lucida* was collected in the fields of the Barrolândia station of CEPLAC in Belmonte, state of Bahia, Brazil (47°73'02"S, 82°21'24"W). The pupa was dissected and the cerebral ganglia were used for obtaining mitotic metaphases according to Imai et al. (1988). Metaphases were stained using Giemsa stain (1:30) and analyzed with an Olympus BX60 microscope equipped with a digital camera. The adult specimen and pupa were studied and photographed with an Olympus SZX7 stereomicroscope. The photographs of the collected insect specimens and metaphases were taken using the Image Pro Plus® version 4.1 analysis software (Media Cybernetics). Karyotypes were digitally mounted and the chromosomes were grouped according to Levan et al. (1964).

## Results and discussion

**Figure 2.**
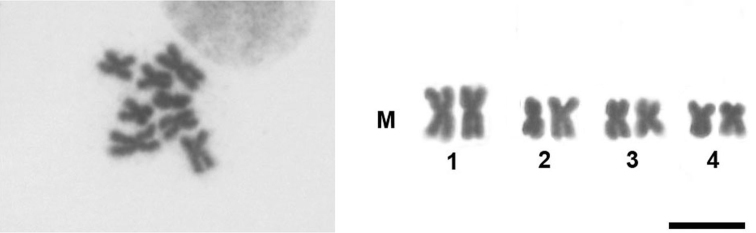
Metaphase plate and diploid karyotype of *Kapala* sp. Bar=10 µm.

This is the first description of the karyotype of a eucharitid wasp. A total of four metaphases were obtained, all of them showing 2n=8 chromosomes. The karyotype of this species (Fig. 2) showed four pairs of metacentric chromosomes with the first pair longer than the remaining three. This karyotype pattern is similar to others described in parasitic wasps with a low chromosome number (n=3–5) (Gokhman and Gumovsky 2009). Considering the known phylogenetic relationships, Eucharitidae together with Perilampidae and Pteromalidae belong to a derived family group of parasitic wasps that probably has undergone independent reductions in chromosome number during karyotype evolution (Gokhman and Gumovsky 2009, Murray et al. 2013). These assumptions are based on the observations that the haploid chromosome number n=11 could be an ancestral character state for the superfamily Chalcidoidea (Gokhman 2013). Species with a low chromosome number are assumed to have undergone chromosome fusions, which would lead to the reduction in chromosome number and consequently to an increase in chromosome size.

The chromosome number of n=4 in *Kapala* sp. (Eucharitidae) is quite similar to those reported in other Chalcidoidea families such as Perilampidae (n=3) and Pteromalidae (n=4–7). *Perilampus ruschkai* Hellén (Perilampidae), n=3 (Gokhman 2005), was clustered in the same clade with Eucharitidae and some Pteromalidae subfamilies in a phylogenetic tree of Chalcidoidea (Gokhman and Gumovsky 2009). According to the latter paper, in the clade comprising Eucharitidae, Perilampidae, and Pteromalidae, a reduction in chromosome number from n=9–11 to n=3–7 during karyotype evolution occurred independently from other clades such as Trichogrammatidae + Eulophidae and Chalcididae + Leucospidae. As discussed in Gokhman and Gumovsky (2009) and Munro et al. (2011), Perilampidae (even if sometimes recovered as a paraphyletic clade) is the sister group of Eucharitidae. According to Gokhman (2011), although studies on the evolution of karyotypes in parasitic wasps are still scarce, it is known that the chromosome rearrangements involved in events of karyotype evolution are mainly fusions (centric and tandem), pericentric inversions, and rare cases of fission followed by the growth of constitutive heterochromatin. This author also points out that similar karyotypes have been described in groups somewhat distinct taxonomically, a phenomenon called karyotypic orthoselection by White (1973).

The karyotype of *Kapala* sp. analyzed herein falls into the seventh karyotype pattern group defined by Gokhman (2011), in which n values vary from 3-7, with a metacentric: acrocentric ratio ≥ 1 and the first chromosome pair less than 1.5 times longer than the remaining three pairs. According to Gokhman (2011), this karyotype pattern represents the final stage of pairwise chromosomal fusions for parasitoid wasps. This first description of the karyotype of a *Kapala* species corroborates a previous hypothesis that this group of wasps would have a low-numbered karyotype (Gokhman and Gumovsky 2009) and therefore confirms that multiple parallel chromosomal fusions have taken place in several lineages within the superfamily Chalcidoidea (Gokhman 2013). Further analyses including larger samples and other cytogenetic techniques will provide more information for the better understanding of the role of chromosomal rearrangements in the evolution of parasitoid wasps.

## References

[B1] Buys SC Cassaro R Salomon D (2010) Biological observations on *Kapala* Cameron 1884 (Hymenoptera Eucharitidae) in parasitic association with *Dinoponera lucida* Emery 1901 (Hymenoptera Formicidae) in Brazil. Tropical Zoology 23: 29–34. http://www.fupress.net/index.php/tropicalzoology/article/viewFile/8659/8077

[B2] Fusu L (2008) The usefulness of chromosomes of parasitic wasps of the subfamily Eupelminae (Hymenoptera: Chalcidoidea: Eupelmidae) for subfamily systematics. European Journal of Entomology 105: 823–828. doi: 10.14411/eje.2008.109

[B3] Gokhman VE (2005) New Chromosome Records for the Superfamily Chalcidoidea (Hymenoptera). Cytologia 70: 239–241. doi: 10.1508/cytologia.70.239

[B4] Gokhman VE (2009) Karyotypes of Parasitic Hymenoptera. Dordrecht, XIII+183 pp. doi: 10.1007/978-1-4020-9807-9

[B5] Gokhman VE (2011) Morphotypes of chromosomes sets and pathways of karyotype evolution of parasitic Hymenoptera. Russian Entomological Journal 20: 265–271. http://zmmu.msu.ru/files/images/spec/Russ%20Ent%20J/ent20_3%20265_271%20Gokhman.pdf

[B6] Gokhman VE (2013) Parallel pathways of karyotype evolution in the superfamilyChalcidoidea (Hymenoptera). Russian Entomological Journal 22(3): 177–179. http://www.kmkjournals.com/upload/PDF/REJ/22/ent22_3%20177_179%20Gokhman.pdf

[B7] Gokhman VE Gumovsky AV (2009) Main trends of karyotype evolution in the superfamily Chalcidoidea (Hymenoptera). Comparative Cytogenetics 3: 63–69. doi: 10.3897/compcytogen.v3i1.1 10.3897/CompCytogen.v14i3.56535 PMC9849058 36761105

[B8] Heraty JM (2002) A revision of the genera of Eucharitidae (Hymenoptera: Chalcidoidea) of the World. Memoirs of the American Entomological Institute 68: 1–367.

[B9] Heraty JM Burks RA Cruaud A Gibson GAP Liljeblad J Munro J Rasplus JY Delvare G Janšta P Gumovsky A Huber J Woolley JB Krogmann L Heydon S Polaszek A Schmidt S Darling DC Gates MW Mottern J Murray E Molin AD Triapitsyn S Baur H Pinto JD Noort S George J Yoder M (2013) A phylogenetic analysis of the megadiverse Chalcidoidea (Hymenoptera). Cladistics, 466–542. doi: 10.1111/cla.12006 10.1111/cla.12006 34798768

[B10] Imai HT Taylor RW Crosland MW Crozier RH (1988) Modes of spontaneous chromosomal mutation and karyotype evolution in ants with reference to the minimum interaction hypothesis. Japanese Journal of Genetics 63: 159–185. doi: 10.1266/jjg.63.159 3273765 10.1266/jjg.63.159

[B11] Lachaud JP Pérez-Lachaud G (2015) Ectaheteromorph ants also host highly diverse parasitic communities: a review of parasitoids of the Neotropical genus *Ectatomma*. Insectes Sociaux 62: 121–132. doi: 10.1007/s00040-015-0390-x

[B12] Lachaud JP Cerdan P Pérez-Lachaud G (2012) Poneromorph ants associated with parasitoid wasps of the genus *Kapala* Cameron (Hymenoptera: Eucharitidae) in French Guiana. Psyche 2012: 1–6. doi: 10.1155/2012/393486

[B13] Levan A Fredga K Sandberg AA (1964) Nomenclature for centromeric position on chromosomes. Hereditas 52: 201–220. doi: 10.1111/j.1601-5223.1964.tb01953.x

[B14] Munro JB Heraty JM Burks R Hawks D Mottern J Cruaud A Rasplus J-Y Jansta P (2011) A molecular phylogeny of the Chalcidoidea (Hymenoptera). PLoS ONE 6: 1–27. doi: 10.1371/journal.pone.0027023 10.1371/journal.pone.0027023 PMC3207832 22087244

[B15] Murray EA Carmichael AE Heraty JM (2013) Ancient host shifts followed by host conservatism in a group of ant parasitoids. Proceedings of the Royal Society B 280: . doi: 10.1098/rspb.2013.0495 10.1098/rspb.2013.0495 PMC3619522 23554396

[B16] Noyes JS (2014) Universal Chalcidoidea Database. http://www.nhm.ac.uk/chalcidoids [accessed 7 September 2014]

[B17] Pérez-Lachaud G Heraty JM Carmichael A Lachaud JP (2006) Biology and behavior of *Kapala* (Hymenoptera: Eucharitidae) attacking *Ectatomma*, *Gnamptogenys*, and *Pachycondyla* (Formicidae: Ectatomminae and Ponerinae) in Chiapas, Mexico. Annals of the Entomological Society of America 99: 567–576. doi: 10.1603/0013-8746(2006)99[567:BABOKH]2.0.CO;2

[B18] White MJD (1973) Animal cytology and evolution. Cambridge, 961 pp.

